# Supplementary material for: Varroa destructor infestation impairs the improvement of landing performance in foraging honeybees
Source: R Soc Open Sci. 2020 Sep 9;7(9):201222. doi: 10.1098/rsos.201222 (PMC7540786; doi:10.1098/rsos.201222)
Supplement: supplementary material [file rsos201222supp1.pdf]

Electronic supplementary material of

Varroa destructor impairs the improvement of landing performance in foraging honeybees

Florian T Muijres[1*], Coby van Dooremalen[2], Martin Lankheet[1], Heleen Lugt[1], Lana J. de Vries[1,2,3,4], Frank van Langevelde[3]

[1] Experimental Zoology Group, Wageningen University & Research, The Netherlands

[2] Bees@WUR, Wageningen University & Research, The Netherlands

[3] Wildlife Ecology and Conservation Group, Wageningen University & Research, The Netherlands

[4] Behavioural Ecology Group, Wageningen University & Research, The Netherlands

[*] Corresponding author: florian.muijres@wur.nl

Consisting of:

- List of symbols and abbreviations
- Supplementary methodology
- Figures S1-S2
- Tables S1–S4
- Legends of Movies S1–S2 and Database S1.

List of symbols and abbreviations

abbreviations	description
DF	Degrees-of-Freedom
DLT	Direct Linear Transformation
GLMM	Generalized Linear Mixed-effects Model
IR	Infra-Red
LED	Light Emitting Diode
UV	Ultra-Violet
V–	non-*Varroa* infected colonies (control)
V+	*Varroa*-infected colonies

symbols	unit	description
$\mathbf{A} = \{a_x, a_y, a_z\}$	m/s^2	acceleration vector
a_x	m/s^2	acceleration normal to the landing platform
f	-	function
z	m	height relative to landing point
I	-	video frame at maximum a_x
i	-	video frame number
L	-	binomial leg extension
l	m	leg extension
l_0	m	leg length
N	-	number of landings
P	-	chance
p	-	probability value
T	ºC	Air temperature
t	-	GLMM test statistics
t	s	time relative to touchdown (first contact)
t_{day}	h	Time-of-day
$\mathbf{U} = \{u, v, w\}$	m/s	velocity vector
V	m/s	speed scalar
V_{approach}	m/s	mean approach flight speed
$\mathbf{X} = \{x, y, z\}$	m	position vector relative to the landing point
Δt	s	time difference
ΔV	m/s	speed change
$\Delta V_{\mathrm{impact}}$	m/s	speed change at impact
$\Delta V_{\mathrm{required}}$	m/s	speed change required for landing
$\Delta V^*_{\mathrm{impact}}$	-	speed change at impact relative to approach speed
ρ	-	linear correlation coefficient

Supplementary methodology

A mechanistic model for predicting landing success

To study how landing success is related to variations in flight kinematics throughout the landing manoeuvre, we developed a biomechanical mechanistic model for predicting the probability of making a successful landing based on the kinematics of the landing manoeuvre.

To successfully land, a bee needs to reduce its momentum at free flight down to zero after touch-down [16]. Flying animals can achieve this using three mechanisms [21]: (1) the animal can perform in-flight aerodynamic braking by producing an aerodynamic force vector in the direction opposite to its flight speed; (2) the animal can convert kinetic energy into potential energy by gaining height during the approach; and (3) the animal can reduce the momentum left at touchdown by absorbing the associated kinetic energy using the leg's muscle-tendon-cuticle system (i.e. the landing gear system). We expect that successful landings can be characterized by aerodynamic braking in combination with height gain during flight, and leg extension prior to touchdown in order to maximize energy absorbance of the landing system at impact. If the legs cannot fully absorb the momentum at touch-down the animal will bounce back, and the landing will fail.

Based on this, we here assume that the probability of making a successful landing decreases with increasing approach speed, because a high approach speed requires a larger speed reduction throughout the landing; landing success also decreases with speed change at impact, as this puts a higher load on the landing system; finally, landing success increases with relative leg extension, as extended legs have a higher energy absorption capacity. Given this, we expressed the probability of landing successfully as a function of the approach and landing kinematics as

$$P(\text{success}) = f(V_{\text{approach}}, \Delta V_{\text{impact}}, l/l_0), \qquad \text{Eq. S1}$$

where P(success) is the probability of making a successful landing; V_{approach} is the approach flight speed, which equals the speed change required for a successful landing (V=0 m/s after touchdown); ΔV_{impact} is the required speed change at impact; l/l_0 is relative leg-extension, where l equals leg extension at first contact, and l_0 is leg length at maximum extension.

To develop and test this model for landing success, the flight kinematics metrics of the model (Eq. 1) needed to be estimated based on the available landing kinematics parameters. We estimated the approach flight speed (V_{approach}) as the average flight speed throughout the landing manoeuvre. The approach phase started when a bee entered a sphere of 50 mm around the position of touchdown and ended at landing (figure 1*d,e*). The sphere of 50 mm radius was chosen such that more than 85% of the analysed trajectories extended to beyond that sphere, and thus V_{approach} could be determined for this subset.

The speed change at impact ΔV_{impact} was determined by numerically integrating the acceleration of the bee for three video frames centred around the moment of impact as

$$\Delta V_{\text{impact}} = \sum_{i=I-1}^{i=I+1} a_x(i)\, \Delta t, \qquad \text{Eq. S2}$$

where a_x is the acceleration of the bee in the direction normal to the landing platform, i is video frame, I is the video frame at which a_x was maximum, and Δt is the temporal resolution of the video (3.3 ms).

We ignored accelerations tangential to the platform, because at impact accelerations normal to the platform cause the largest and most relevant velocity change.

Because landing manoeuvres with a high approach speed V_{approach} tend to also have high speed changes at impact ΔV_{impact}, these two parameters were highly correlated (Figure S1A, linear correlation coefficient ρ=0.80, p<0.001). This makes it impossible to discriminate between their relative effects on landing success. To solve this, we normalized ΔV_{impact} with V_{approach} by

$$\Delta V^*_{\text{impact}} = \frac{\Delta V_{\text{impact}}}{V_{\text{approach}}}. \qquad \text{Eq. S3}$$

The resulting relative speed changes at impact $\Delta V^*_{\text{impact}}$ was not significantly correlated with V_{approach} (Figure S1B, ρ=-0.08, p=0.064); it expresses the part of the total required speed change that was achieved by breaking at impact using its leg system. As discussed in the main text, leg extension at touchdown was scored binomial (L), and thus our functional model for landing success based on the measured flight kinematics is

$$P(\text{success}) = f(V_{\text{approach}}, \Delta V^*_{\text{impact}}, L). \qquad \text{Eq. S4}$$

Supplementary figures

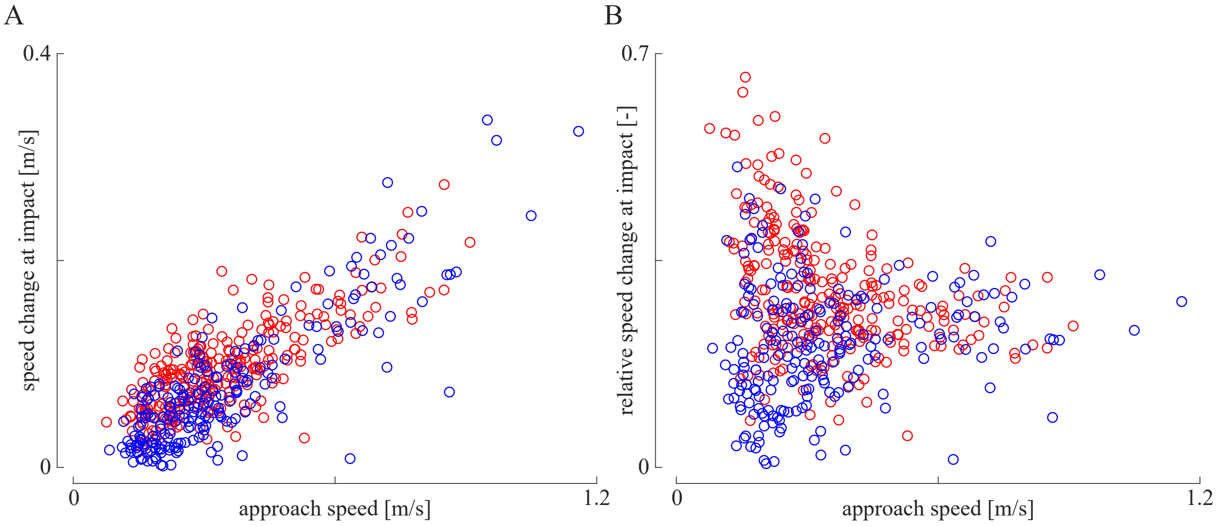

Figure S1. Speed change at impact, relative speed change at impact and approach speed for all analysed landing manoeuvres (*N*=486 landings). Data for successful and failed landings are in blue and red, respectively. (A) Speed change at impact correlates strongly with approach speed (linear correlation coefficient ρ=0.80, p<0.001); (B) Relative speed change at impact does not correlate significantly with approach speed (ρ=-0.08, p=0.064).

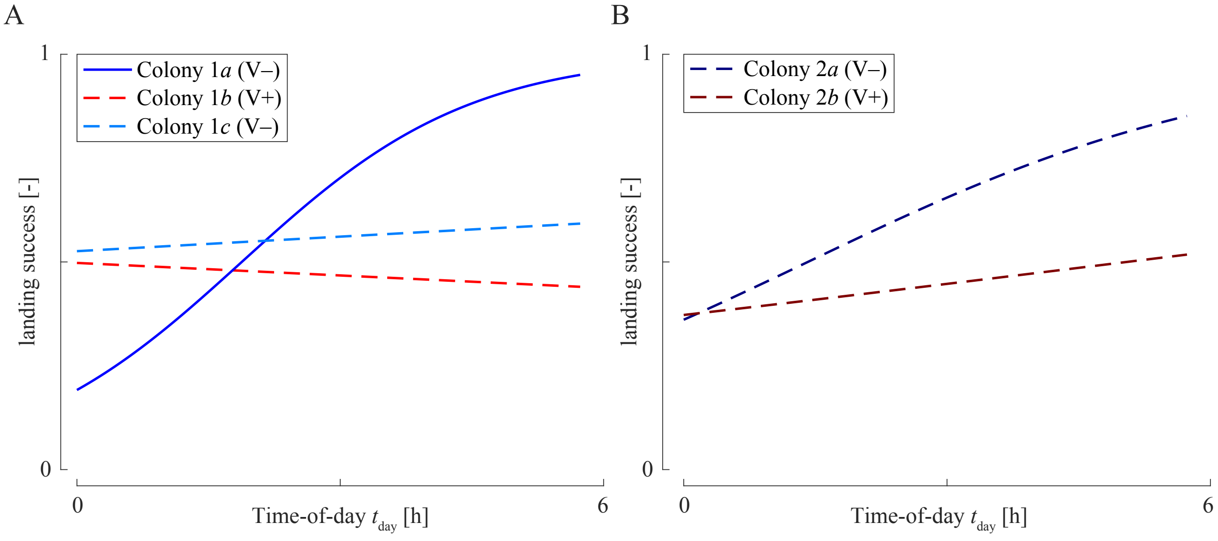

Figure S2. The temporal dynamics of landing success probability throughout the experimental days for each tested colony separately. Panel A shows data for the paired sister-colonies 1*a*, 1*b* and 1*c*; panel B shows data for the paired sister-colonies 2*a* and 2*b* (see the legends in A and B for details). The trend lines show predicted success probability estimated using GLMM's for each colony separately (Table S2). Solid and dashed trendlines show a significant and a non-significant effect of time-of-day on landing success, respectively.

Supplementary tables

Table S1. Experimental database, including date of each experiment, hive number, mite load and amount of analysed landing manoeuvres. Colonies with the same numbers are paired colonies, whereby the extensions *a* and *b* refer to colonies with sister queens. Colony 1*c* is a replacement of colony 1*a*.

Experimental Date	Colony number	Mite load [mites/100 bees]	Number of landings
July 5th	1*a*	0	61
July 6th	1*b*	1.3	58
July 9th	2*a*	0	55
July 10th	2*b*	0.9	94
July 11th	3*a*	0	61
July 12th	3*b*	0	21
August 21st	2*b*	2.1	51
August 22nd	2*a*	0	42
August 23rd	1*b*	1.9	30
August 24th	1*c*	0	16
August 27th	3*b*	0	24
August 28th	3*a*	0	42

Table S2. GLMM results of the test on how landing success varies throughout the experimental day and between bees from the paired *Varroa*-infested colonies and control colonies. Results are given for the complete model, and for the post-hoc tests on the *Varroa*-infested colonies (V+), the control colonies (V–), and each colony separately. Significant *p*-values are highlighted in bold.

**Success rate**	**Estimate**	**SE**	**t**	**DF**	***p*-value**
Time-of-day	0.423	0.149	2.848	403	**0.005**
Varroa	0.539	0.433	1.245	403	0.213
Time-of-day × *Varroa*	-0.403	0.176	-2.282	403	**0.023**

**Success rate (V– post-hoc)**	**Estimate**	**SE**	**t**	**DF**	***p*-value**
Time-of-day	0.424	0.149	2.848	172	**0.005**

**Success rate (V+ post-hoc)**	**Estimate**	**SE**	**t**	**DF**	***p*-value**
Time-of-day	0.026	0.095	0.268	231	0.789

**Success rate (Colony 1*a*, V– post-hoc)**	**Estimate**	**SE**	**t**	**DF**	***p*-value**
Time-of-day	0.766	0.293	2.615	59	**0.011**

**Success rate (Colony 1*b*, V+ post-hoc)**	**Estimate**	**SE**	**t**	**DF**	***p*-value**
Time-of-day	-0.040	0.177	-0.227	86	0.821

**Success rate (Colony 1*c*, V– post-hoc)**	**Estimate**	**SE**	**t**	**DF**	***p*-value**
Time-of-day	0.047	0.342	0.137	14	0.893

**Success rate (Colony 2*a*, V– post-hoc)**	**Estimate**	**SE**	**t**	**DF**	***p*-value**
Time-of-day	0.404	0.233	1.733	95	0.086

**Success rate (Colony 2*b*, V+ post-hoc)**	**Estimate**	**SE**	**t**	**DF**	***p*-value**
Time-of-day	0.103	0.118	0.876	143	0.382

Table S3. GLMM results of the test on how landing success depends on the flight kinematics parameters pre-touchdown leg extension (*L*), approach speed ($V_{approach}$), and relative speed change at impact (ΔV^*_{impact}). Results are given after iteratively removing the least significant interactions from the model. Significant *p*-values are highlighted in bold.

Success rate	Estimate	SE	t	DF	*p*-value
L	3.025	0.981	3.082	480	**0.002**
$V_{approach}$	-3.055	1.354	-2.256	480	**0.025**
ΔV^*_{impact}	-2.224	1.758	-1.265	480	0.206
$L \times V_{approach}$	3.994	1.651	2.420	480	**0.016**
$L \times \Delta V^*_{impact}$	-5.695	2.655	-2.145	480	**0.032**

Table S4. GLMM results of the test on how pre-touchdown leg extension (*L*), approach speed ($V_{approach}$), and relative speed change at impact (ΔV^*_{impact}) varied with time-of-day, and between the paired *Varroa*-infested colonies and control colonies. Results are given after iteratively removing the least significant interactions from the model. For ΔV^*_{impact} we also show results of the post-hoc tests on the paired *Varroa*-infested colonies (V+) and control colonies (V−), separately. Significant *p*-values smaller than 0.05 are highlighted in bold.

pre-touchdown leg extension	Estimate	SE	t	DF	*p*-value
Time-of-day	0.208	0.081	2.577	404	**0.010**
Varroa	-0.466	0.223	-2.093	404	**0.037**

Approach speed	Estimate	SE	t	DF	*p*-value
Time-of-day	-0.002	0.008	-0.285	350	0.776
Varroa	0.006	0.028	0.212	350	0.832

ΔV^*_{impact}	Estimate	SE	t	DF	p-value
Time-of-day	-0.040	0.008	-4.697	349	**<0.001**
Varroa	-0.076	0.025	-3.022	349	**0.003**
Time-of-day × *Varroa*	0.042	0.010	4.118	349	**<0.001**

ΔV^*_{impact} (V− post-hoc)	Estimate	SE	t	DF	p-value
Time-of-day	-0.040	0.009	-4.356	145	**<0.001**

ΔV^*_{impact} (V+ post-hoc)	Estimate	SE	t	DF	p-value
Time-of-day	0.002	0.005	0.436	204	0.663

Supplementary movie and database legends

Movie S1. Stereoscopic high-speed video of a failed landing attempt of a bee that did not extend its legs prior to touchdown. The stereo video consists of a side camera recording (left) and bottom camera recording (right). See Figure 1*a* for the setup. Cameras were synchronized and recorded at 300 frames per second; playback was slowed down 10 times. Analysis results of this video are reported in Figure 1*b,d-g*.

Movie S2. Stereoscopic high-speed video of a successful landing manoeuvre of a bee that extended its legs prior to touchdown. The stereo video consists of a side camera recording (left) and bottom camera recording (right). See Figure 1*a* for the setup. Cameras were synchronized and recorded at 300 frames per second; playback was slowed down 10 times. Analysis results of this video are reported in Figure 1*c-g*.

Database S1. Database with the data supporting this study, stored in a MATLAB (MathWorks, Inc.) structured binary data container format. The database consists of two datasets: 1. The structure "trajectories" contains the temporal dynamics of all analysed trajectories, including the time array t [s], and vectors of position $\mathbf{X} = \{x,y,z\}$ [mm], velocity $\mathbf{U} = \{u,v,w\}$ [m/s], and accelerations $\mathbf{A} = \{a_x,a_y,a_z\}$ [m/s^2] of the bee throughout each landing manoeuvre. 2. The table "landing_metrics_and_meta_data" contains all derived metrics and meta-data used for our GLMM tests. It includes for each analysed landing manoeuvre the experimental day [yyyymmdd], Time-of-day [h], Hive number [-], *Varroa*-infestation of the colony V–/V+ [-], Pre-touchdown leg extension L [-], Approach speed V_{approach} [m/s], and Relative speed change at impact $\Delta V^*_{\text{impact}}$ [-].
